# Long noncoding RNA and mRNA profiling in cetuximab‐resistant colorectal cancer cells by RNA sequencing analysis

**DOI:** 10.1002/cam4.2004

**Published:** 2019-03-07

**Authors:** Changwen Jing, Rong Ma, Haixia Cao, Zhuo Wang, Siwen Liu, Dan Chen, Yang Wu, Junying Zhang, Jianzhong Wu

**Affiliations:** ^1^ Clinical Cancer Research Center Jiangsu Cancer Hospital & Jiangsu Institute of Cancer Research & The Affiliated Cancer Hospital of Nanjing Medical University Nanjing Jiangsu Province China

**Keywords:** cetuximab, colorectal cancer, lncRNA, mRNA, RNA‐Seq

## Abstract

To gain an insight into the molecular mechanisms of cetuximab resistance in colorectal cancer, we generated a cetuximab‐resistant cell line (H508/CR) and performed RNA sequencing to identify the differential expression patterns of noncoding RNAs (ncRNAs) and mRNAs between cetuximab‐sensitive and resistant cells. A total of 278 ncRNA transcripts and 1,059 mRNA transcripts were dysregulated in the cetuximab‐resistant cells. The expression levels of nine selected long noncoding RNAs (lncRNAs) were validated using quantitative real‐time PCR. Functional analysis revealed that several groups of lncRNAs might be involved in pathways associated with cetuximab resistance. Increased glucose consumption and lactate secretion in cetuximab‐resistant cells suggested that glucose metabolism might be involved in cetuximab resistance. In addition, lncRNA *LINC00973* was upregulated in the H508/CR cell line and cells transfected with a *LINC00973* short interfering RNA exhibited reduced cell viability, increased apoptosis, and decreased glucose consumption and lactate secretion. Our results provide essential data regarding differentially expressed lncRNAs and mRNAs in cetuximab‐resistant cells, which may provide new potential candidates for cetuximab therapy.

## 1 INTRODUCTION

Colorectal cancer (CRC) is one of the leading causes of cancer‐related death throughout the world.^1^ Cetuximab is an epidermal growth factor receptor (EGFR) monoclonal antibody that acts by blocking EGFR's extracellular domain, thereby negatively affecting cell growth and producing an antitumor effect. For patients with metastatic CRC (mCRC), the combined application of cetuximab and conventional chemotherapy is a typical therapeutic regimen that can reduce the risk of progression, improving response rates by up to 72% compared with cetuximab monotherapy.^2^ The National Comprehensive Cancer Network (NCCN) guidelines recommend detecting mutations in the genes encoding KRAS (V‐Ki‐Ras2 Kirsten rat sarcoma viral oncogene homolog) proto‐oncogene, GTPase (KRAS), NRAS (neuroblastoma RAS viral oncogene homolog) proto‐oncogene, GTPase (NRAS), and B‐raf proto‐oncogene, serine/threonine kinase (BRAF) to predict the efficacy of cetuximab. However, some patients with wild‐type *KRAS*,* NRAS*, and *BRAF* acquire resistance to cetuximab after an initial period of treatment.^3^ However, the nongenetic mechanism of research on acquired cetuximab resistance is scarce.

Over the past decade, advances in high‐throughput RNA sequencing (RNA‐Seq) technologies and bioinformatic methods have facilitated the study of the entire human genome sequence, including noncoding RNAs (ncRNA), such as microRNAs and long noncoding RNAs (lncRNAs).^4^, ^5^ LncRNAs are noncoding transcripts longer than 200 nucleotides, which play important roles in a variety of biological processes, involving chromatin modification, transcriptional regulation, posttranscriptional processing, RNA editing, cell cycle regulation, alternative splicing, and organelle biogenesis.^6^ Many studies have confirmed that lncRNAs are closely related to drug sensitivity, for instance lncRNA *UCA1* promotes oral squamous cancer cell proliferation and cisplatin resistance by inhibiting the activity of microRNA miR‐184.^7^ *LncARSR* promotes the expression of the AXL receptor tyrosine kinase (AXL) and MET proto‐oncogene, receptor tyrosine kinase (c‐MET) by competitively binding to miR‐34/miR‐449 to induce sunitinib resistance in renal cancer cells.^8^

In our study, we generated a cetuximab‐resistant cell line (NCI‐H508/CR) and performed RNA‐Seq to identify the differential expression patterns of lncRNAs and mRNAs between cetuximab‐sensitive and resistant cells. Quantitative RT‐PCR was applied to verify the RNA‐Seq data. We constructed the lncRNA‐gene network using specific bioinformatic analyses to explore potential lncRNAs associated with cetuximab resistance, which may provide potential candidates for CRC treatment.

## 2 MATERIALS AND METHODS

### 2.1 Cell culture

We selected cetuximab‐sensitive NCI‐H508 cell line (epithelial cells from colorectal adenocarcinoma) from the ATCC (Cat. no. CCL‐253; Manassas, VA, USA) based on the study of Medico et al^9^ The cells were maintained in Roswell Park Memorial Institute (RPMI)‐1640 medium (ATCC^®^ 30‐2001^™^) supplemented with 10% fetal bovine serum (FBS), 100 U/mL penicillin, and 100 mg/mL streptomycin in a 37°C incubator with 5% CO_2_. SW480 (ATCC cat. no. CCL‐228), DLD‐1 (ATCC cat. no. CCL‐221), and LoVo (ATCC cat. no. CCL‐229) human CRC cell lines were obtained from Professor Wang of the Nanjing medical university. The three cell lines were cultured in Dulbecco's modified Eagle's medium with 10% FBS, 100 U/mL penicillin, and 100 mg/mL streptomycin under standard culture conditions.

### 2.2 Generation of cetuximab‐resistant NCI‐H508 cells in vitro

Cetuximab was purchased from Merck (Imported drug registration number: S20130004, Darmstadt, Germany). To generate the resistant cell line, NCI‐H508 cells were treated with increasing concentrations of cetuximab, starting with the IC_50_ concentration (the drug concentration inducing 50% cell growth inhibition). To verify the lasting effect of cetuximab resistance, the obtained NCI‐H508 cells were cultured without cetuximab for 8 weeks and then tested using Cell Counting Kit‐8 assays (CCK8) (cat. no. CK04; Dojindo Molecular Technologies, Inc., Kumamoto, Japan). The sensitive and resistant NCI‐H508 cells were named H508S and H508/CR, respectively.

### 2.3 Cell viability assay

The CCK8 assay was used to evaluate cell viability. The cells were seeded at approximately 5 × 10^3^ cells per well in a 96‐well plate and treated with different concentrations of cetuximab after 24 hours of cell attachment. After 72 hours, CCK8 reagents were added into the wells and the OD values (absorbance) were measured at 450 nm using a Microplate Reader (BioTek ELx800; BioTek Instruments Inc., Winooski, VT, USA). The results were converted as previously described.^10^

### 2.4 Mutation detection of cells using an Ion torrent Personal Genome Machine^™^

Mutation detection was performed as previously described.^11^ Briefly, after DNA extraction from H508/S and H508/CR cells and concentration determination, 15 ng of DNA was used for library construction. Following library enrichment, the products were sequenced on an Ion torrent Personal Genome Machine^™^. The sequences carrying mutations were compared with human genome release hg19.

### 2.5 Next generation RNA sequencing analysis

Next generation RNA‐Seq analysis was conducted by RiboBio Co., Ltd (Guangzhou, China). In brief, total RNAs were isolated separately from H508S and H508/CR cells using the TRIzol reagent (Cat. no. 15596026; Thermo Fisher Scientific Inc., Austin, TX, USA). The RNA concentration and quality were evaluated using a NanoDrop ND1000 spectrophotometer (NanoDrop, Wilmington, DE, USA) and an Agilent 2100 Bioanalyzer (Agilent Technologies, Santa Clara, CA, USA), respectively. The libraries were constructed with 1 μg of RNA with a NEBNext^®^ Ultra^™^ RNA Library Prep Kit for Illumina (NEB, Ipswich, MA, USA; Cat. no. #E7420). The Illumina HiSeq^™^ 3000 platform (Illumina, San Diego, CA, USA) was used for RNA‐Seq.

### 2.6 Flow cytometry analysis

H508/CR cells were plated at 1 × 10^6^ cells/well in a six‐well plate and stimulated with or without cetuximab. Seventy‐two hours later, the cells were stained using a fluorescein isothiocyanate (FITC)/Annexin V apoptosis detection kit (Cat. no. 556547; BD Biosciences, San Diego, CA, USA). Then, the samples were loaded onto a flow cytometer (C6; Becton Dickinson, San Diego, CA, USA).

### 2.7 qRT‐PCR assay

One‐microgram of RNA was reverse transcribed into cDNA using a Takara PrimeScript^™^ RT Master Mix kit (cat. no. RR036Q; Takara Bio Inc., Otsu, Japan). The PowerUp^™^ SYBR^®^ Green Master Mix (Cat.no. A25742; Thermo Fisher Scientific Inc.) and an Applied Biosystems 7300 Real‐Time PCR System (Applied Biosystems, Foster City, CA, USA) were used for the qRT‐PCR assay. The PCR was performed under the following conditions: (a) 94°C for 30 seconds; (b) 40 cycles of 94°C for 5 seconds and 60°C for 30 seconds; and (c) 95°C for 1 minute, 55°C for 30 seconds, and then 95°C for 30 seconds. Three technical replicates of each PCR reaction were carried out and *ACTB* (encoding β*‐*actin) was selected as a house‐keeping gene. The primers for the lncRNAs were designed and purchased from RiboBio Co. Ltd. The primers for the mRNAs were purchased from Sangon Biotech Co., Ltd (Shanghai, China). All the primers are described in Table S1. The relative RNA levels were determined using the …Ct method relative to the internal control gene.^12^

### 2.8 Extracellular glucose and lactate levels measurement

Cells (1 × 10^5^) were seeded wells of 24‐well plates and cultured for 24 hours. The culture medium was then removed and the cells were incubated for an additional 48 hours. After centrifugation at 1500 *g*, 4°C, for 5 minutes, the lactate and glucose levels in the supernatant were analyzed using an absorbance reader and a Lactic Acid assay kit (Cat. no. A019‐2; Jiancheng Bioengineering Institute, Nanjing, China) and Glucose assay kit (Cat. no. 361500; Rongsheng Biotech Co., Ltd, Shanghai, China), respectively. Three technical replicates were performed and the number of the cells was estimated to normalize the glucose and lactate levels.

### 2.9 Cell transfection and treatment

For transfection, H508/CR cells were placed in six‐well or 96‐well plates. Twenty‐four hours later, they were transfected with Ribo^™^ h‐*LINC00973* Smart Silencer or negative control (NC) (Cat. no. lnc3180815025303; RiboBio Co., Ltd) using Lipofectamine^®^ RNAiMAX reagent (cat. no. 13778075) and Gibco^®^ Opti‐MEM^®^ (cat. no. 31985062) according to the manufacturer's instructions. The target sequences of *LINC00973* short interfering RNA (siRNA) are presented in Table S2. H508/CR cells were treated with or without cetuximab for 72 hours.

### 2.10 RNA fluorescent in situ hybridization (RNA‐FISH)

*LINC00973* fluorescent in situ hybridization was performed using a FISH kit (Cat. no. C10910; RiboBio). H508S and H508/CR cells were seeded on coverslips, which were placed in a 24‐well plate at 1 × 10^5^ cells/well. After 24 hours, they were fixed in 4% paraformaldehyde for 20 minutes, digested for 30 minutes, prehybridized with hybridization solution for 60 minutes at 37°C, and incubated with a FITC‐labeled *LINC00973* probe at 37°C overnight. Cell nuclei were stained with 4′,6‐diamidino‐2‐phenylindole for 5 minutes at room temperature after washing. Finally, the fluorescence images were immediately captured using a Carl Zeiss fluorescence microscope (Gottingen, Gremany).

### 2.11 Statistical analysis

All the figures were prepared using GraphPad Prism 6 software (GraphPad Inc., La Jolla, CA, USA). DESeq v1.18.0 was used in the RNA‐Seq analysis. Gene Ontology (GO) and KEGG pathway analysis were performed using the kobas 3.0 software. The coexpression networks were drawn with Cytoscape software 3.2.0. Statistical analysis was performed using the spss software (version 20.0; IBM Corp., Armonk, NY, USA) and clearly described in the figure legends. The comparison of cell viability and apoptosis between multiple groups was performed using two‐way analysis of variance (ANOVA) followed by Bonferroni posttests. The differences between two groups were analyzed using Student's *t* test. Differences with *P *<* *0.05 were considered statistically significant.

## 3 RESULTS

### 3.1 Establishment of a cetuximab‐resistant cell line

**Figure 1:**
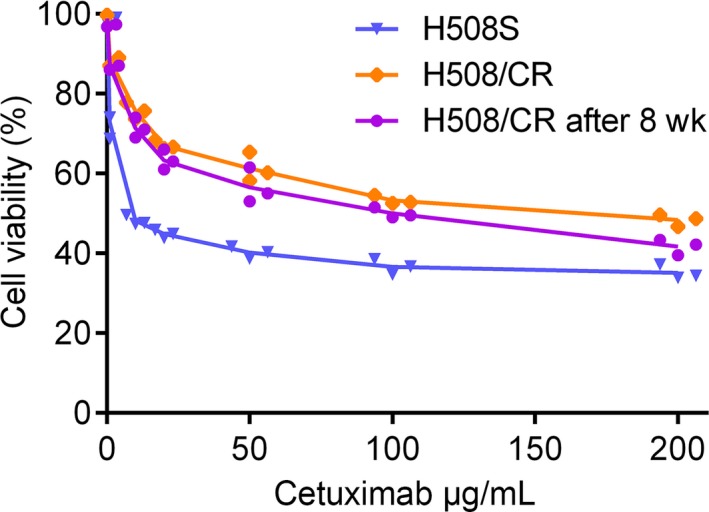
Establishment of cetuximab‐resistant H508/CR cell line. Cells were treated with indicated concentrations of cetuximab (1, 10, 20, 50, 100, 200 μg/mL) for 72 h. Cell viability and IC50 values were determined using CCK‐8 assay. Every CCK‐8 test was carried out in quadruplet. Data are mean ± SD for three independent experiments. Statistical comparisons were performed at each dose using two way ANOVA (group effect, *F*(2,42) = 326.0, *P* < 0.0001) followed by Bonferroni posttests (*P* values were listed in Table S3)

Following continuous exposure to increasing doses of cetuximab for up to 6 months, the H508/CR cell line was generated from the parental H508S cells. Cell viability was determined by the CCK‐8 assay to assess the resistance index of the cetuximab‐resistant cells. The IC_50_ value of H508S for cetuximab was 12.79 ± 2.67 μg/mL. However, the IC_50_ of H508/CR became 154.70 ± 25.67 μg/mL. The resistance index was more than 10‐fold higher. After 8 weeks in the absence of cetuximab, H508/CR cells still maintained strong drug resistance and the IC_50_ value of the cells was 90.51 ± 7.39 μg/mL (Figure 1). There was no significant change in the sensitivity to cetuximab in the parental cells or the derived‐resistant cells during this period.

### 3.2 Mutations of H508S and H508/CR cells

After the resistant cells were successfully established, we performed a next‐generation sequencing (NGS) on H508S and H508/CR cells with a colon and lung panel (genes such as *KRAS, EGFR, BRAF, PIK3CA, ALK, NRAS, ERBB2, MET, MEK1, PTEN, SMAD4, STK11, FBXW7, ERBB4, DDR2, CTNNB1, AKT1, NOTCH1, FGFR1, FGFR2,* and *FGFR3* were included). As shown in Table 1, both H508S and H508/CR cells harbored mutations G596R in *BRAF* and E545K in *PIK3CA*. No previously identified secondary mutations in genes associated with cetuximab resistance were found.

**Table 1:** Gene mutations detected by NGS sequencing

Cell type	Mutant gene	Mutant site	Protein position
H508S	*BRAF* *PIK3CA*	*c.1786G>C* *c.1633G>A*	*p.G596R* *p.E545K*
H508/CR	*BRAF* *PIK3CA*	*c.1786G>C* *c.1633G>A*	*p.G596R p.E545K*

NGS, next‐generation sequencing.

### 3.3 Profiles of the differentially expressed ncRNAs and mRNAs

**Figure 2:**
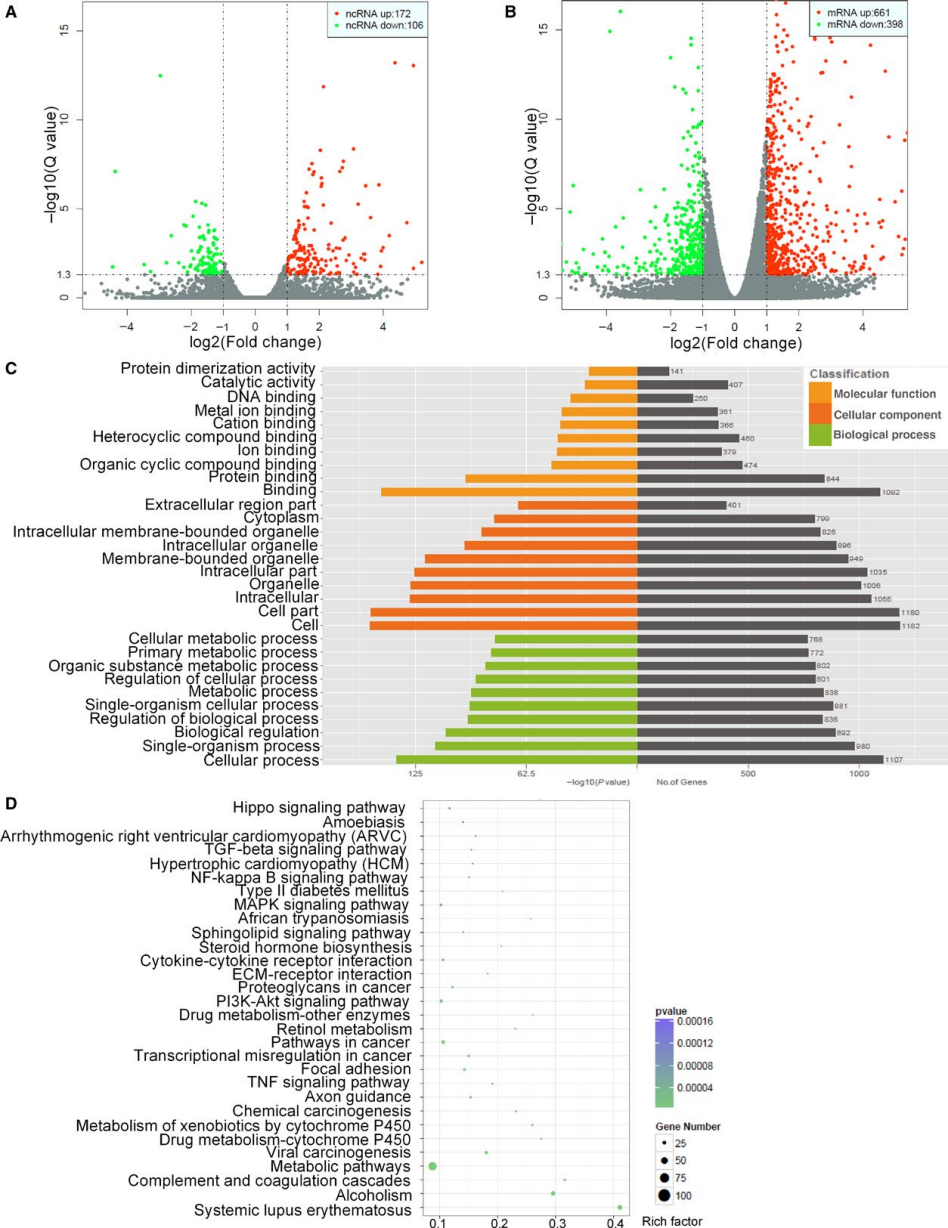
Differential expression of ncRNAs and mRNAs. (A) The volcano map of differential ncRNAs; (B) the volcano map of differential mRNAs. *X*‐axis: the fold change expressed as log2; *Y*‐axis: expressed by *q* value. The vertical dotted lines corresponded to 2.0‐fold up and down, and the horizontal dotted line represented a *Q*‐value of 0.05. The red dots indicates significantly upregulated ncRNAs/genes an green indicates significantly down‐regulated ncRNAs/genes; (C) GO analysis of the differentially expressed genes and the *P* < 0.05 of GO annotation was regarded as the significant threshold; (D) bubble map of KEGG analysis for differentially expressed gene related significant enriched signaling pathway. *X‐*axis in the figure indicated the ratio of the enriched differential gene to the background gene of the pathway. *Y‐*axis showed the name of statistics pathway enrichment. The size of the point in the plot represented the number of enriched differential genes and the color changes from green to purple indicated the *Q‐*value

Next, we focused on RNA levels to further investigate the molecular mechanisms of cetuximab resistance. Therefore, we analyzed ncRNA and mRNA expression profiles in H508S and H508/CR cell lines using RNA‐Seq analysis. The volcano plots generated from the expression of differentially expressed ncRNAs and mRNAs on three pairs of matched H508S and H508/CR showed distinct expression patterns of these ncRNAs and mRNAs between the cetuximab‐sensitive and resistant cell lines (Figure 2A,B).

In H508S and H508/CR cells, 11,889 ncRNA transcripts were detected. Among them, we defined the statistical criteria for selecting aberrantly expressed ncRNAs and mRNAs using a *Q*‐value of <0.05 (to highlight the differences, we used DESeq^13^, ^14^ to adjust the *P* value to get the *Q* value.) with a fold change of >2.0 or <0.5 (|log_2_^FoldChange^|>1). Compared with the transcripts in H508S cells, 278 noncoding transcripts were significantly aberrantly expressed in the cetuximab‐resistant cells, with 172 upregulated and 106 downregulated. A total of 18,881 mRNAs were detected. Six hundred and sixty‐one mRNAs were upregulated and 398 were downregulated significantly in the H508/CR cell line. Tables 2 and 3 list the top 10 upregulated (Table 2) and downregulated mRNAs (Table 3). The expression profiling data suggested the most differentially expressed 20 ncRNAs between the two groups, including the top 10 upregulated (Table 4) and downregulated ncRNAs (Table 5).

**Table 2:** Top 10 upregulated expressed of mRNAs (H508S set as 1)

mRNA	Fold change	Function
*IGFL1*	91	Breast cancer progression (PMID: 28488769)
*FOXL1*	80	Tumor suppressor (PMID: 23801748)
*LMO7*	75	Cell migration (PMID: 21670154)
*HAS2*	65	Oncogene (PMID: 23783513)
*PCP4*	64	Anti‐apoptotic (PMID: PMC5226490)
*MX1*	47	Tumor invasion (PMID: 24771638)
*ZBED2*	42	Diverse function (PMID: 23533661)
*FYN*	40	Oncogene (PMID: 26686094)
*HUNK*	40	Oncogene (PMID: 21393859)
*MX2*	39	Inhibitor of HIV‐1 infection (PMID: 24121441)

**Table 3:** Top10 downregulated expressed of mRNAs (H508S set as 1)

Gene	Fold change	Function
*APOH*	0.0009	Oncogene in hepatocellular carcinoma (PMID: 20204408)
*ALB*	0.0013	Prognostic indicator (PMID: 30119141)
*APOB*	0.0030	Prognostic indicator (PMID: 29713185)
*PLG*	0.0032	Thrombophilia and ligneous conjunctivitis (PMID: 27976734)
*FREM1*	0.0040	Inflammatory response activation (PMID: 19940113)
*ALDOB*	0.0041	Hereditary fructose intolerance (PMID: 29907340)
*FGB*	0.0043	Fibrinogen beta chain (PMID: 29518939)
*ITIH2*	0.0052	Prognostic indicator (PMID: 23704207)
*SERPINC1*	0.0068	Potential cancer markers for intrahepatic cholangiocarcinoma (PMID: 16712791)
*TF*	0.0070	Anticancer therapy (PMID: 24573305)

**Table 4:** Top 10 upregulated expressed of ncRNAs (H508S set as 1)

ncRNA	Gene name	Fold change	RNA type
ENST00000589310.1	*LINC01764*	71	lincRNA
NR_033957.2	*LINC00842*	47	lncRNA
NR_038977.1	*LINC01239*	37	lncRNA
NR_036581.1	*LINC00675*	31	lncRNA
ENST00000430184.1	*AL357033.2*	31	antisense
NR_033807.2	*CYP3A5*	27	misc_RNA
ENST00000473756.1	*LINC00973*	27	lincRNA
NR_135234.1	*IGFL2‐AS1*	26	lncRNA
ENST00000566733.1	*AL590004.4*	21	lincRNA
NR_026975.1	*FIRRE*	20	lncRNA

**Table 5:** Top 10 downregulated expressed of ncRNAs (H508S set as 1)

ncRNA	Gene name	Fold change	RNA type
ENST00000413290.1	*AC104823.1*	0.0392	lincRNA
ENST00000429386.1	*AL121972.1*	0.0456	antisense
NR_024471.1	*MRPL23‐AS1*	0.0480	lncRNA
NR_002994.2	*SNORA36B*	0.0899	snoRNA
NR_003608.1	*TUBA3FP*	0.1037	misc_RNA
ENST00000551799.1	*AL136418.1*	0.1079	antisense
NR_003578.1	*ZNF702P*	0.1281	misc_RNA
NR_003075.1	*SNORD93*	0.1461	snoRNA
ENST00000427584.2	*AC116345.1*	0.1624	lincRNA
NR_033932.1	*RGMB‐AS1*	0.1910	lncRNA

Genes with more than twofold change were further analyzed using bioinformatic tools. The GO (http://www.geneontology.org) functional annotations were for three categories: (a) cell composition: each part of the cell and the extracellular environment; (b) molecular function: the main activity of the gene product at the molecular level, such as binding and catalysis; (c) biological process, providing background knowledge of gene functional classification tags and gene function research (Figure 2C). Biological pathway analysis was used for enrichment analysis to study biological function, based on the biology of the Kyoto Encyclopedia of Genes and Genomes (KEGG) pathways. KEGG pathway analysis demonstrated that significantly differentially expressed genes could have an impact on several cancer‐related pathways, such as metabolic pathways, focal adhesion, pathways in cancer, drug metabolism, the MAP kinase (MAPK) signaling pathway, the NF‐kappa B signaling pathway, and the phosphatidylinositol 3‐Kinase‐AKT (PI3K‐Akt) signaling pathway (Figure 2D).

### 3.4 Real‐time PCR validation and lncRNA function prediction

**Figure 3:**
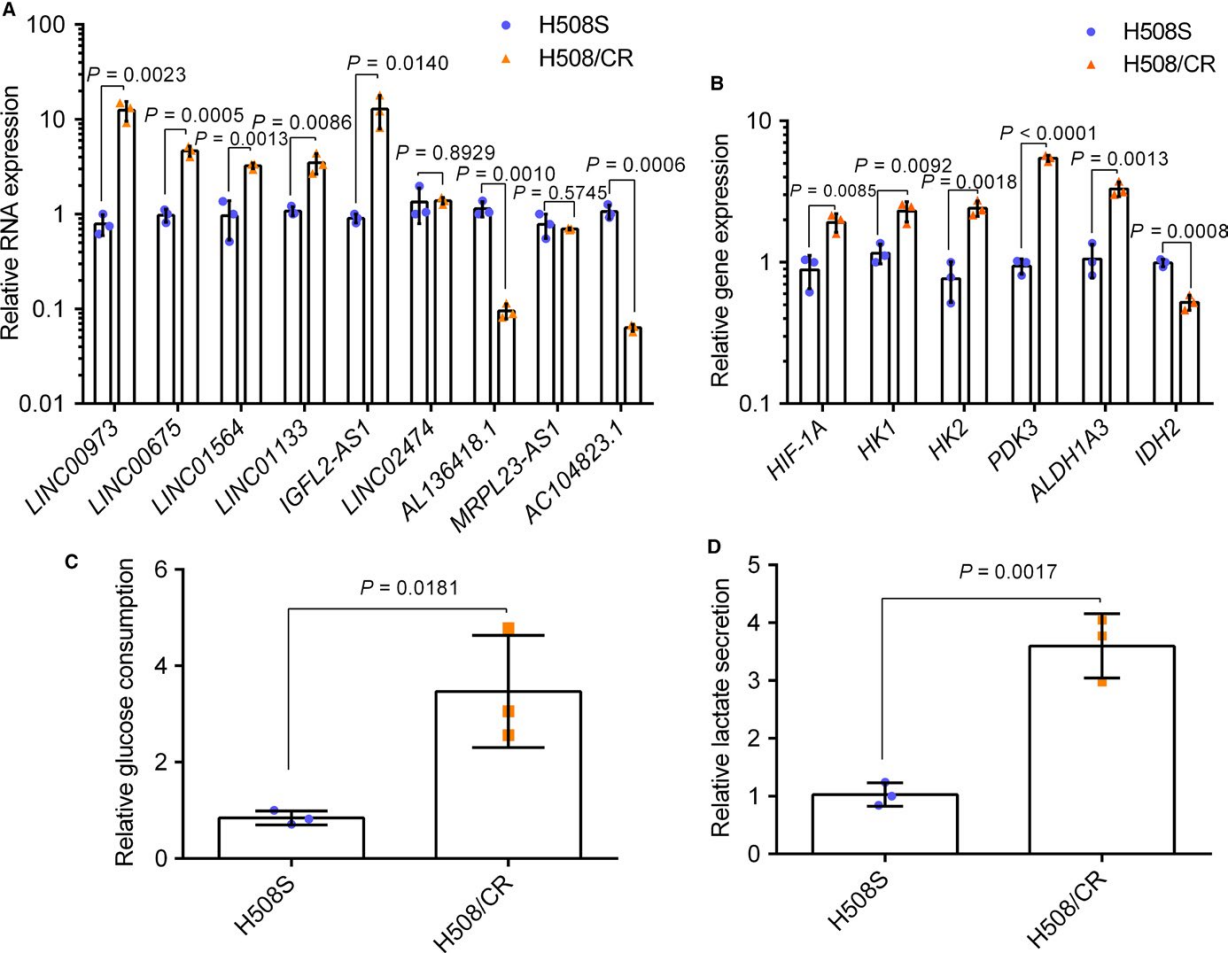
Real‐time PCR validation and lncRNA function prediction. (A) Real‐time quantitative PCR results of nine chosen lncRNAs. The heights of the columns in the chart represent the fold change (H508/CR/H508S cells) in expression for each of these lncRNAs; (B) real‐time quantitative PCR results of glucose metabolism‐related genes. Glucose consumption (C) and secreted lactate levels (D) by H508S and H508/CR in 72 h were shown as fold changes of H508/CR/H508S cells. Unpaired Student's *t* tests were used in the figure. The data were presented in the form of mean ± SD (*n* = 3). Similar results were obtained from three independent experiments. Abbreviation: HIF‐1A, hypoxia‐inducible factor 1‐alpha; HK 1/2, hexokinase 1/2; PDK3, pyruvate dehydrogenase kinase isozyme 3; ALDH1A3, aldehyde dehydrogenase 1 family member A3; IDH2, isocitrate dehydrogenase 2

To validate the dysregulated expression of lncRNAs found by RNA‐seq, we chose six upregulated and three downregulated lncRNAs with RPKM values (expected number of reads per kilobase of transcript sequence per millions base pairs sequenced, which was employed to calculate the expression level of individual lncRNAs) >50. We performed qRT‐PCR to examine the levels of *LINC00973‐201*,* LINC00675*,* IGFL2‐AS1‐203*,* LINC01564*,* LINC01133‐201*,* LINC02474‐202*,* AC104823.1‐201*,* AL136418.1‐201*, and *MRPL23‐AS1* (Figure 3A). The expression trend of seven lncRNAs was consistent with the sequencing results. The expression levels of *LINC00973‐201*,* LINC00675*,* IGFL2‐AS1‐203*,* LINC01564*, and *LINC01133‐201* were increased in H508/CR cells, while *AC104823.1* and *AL136418.1* were decreased, compared with those in the parental H508S cells. The expression levels of *LINC02474‐202* and *MRPL23‐AS1* did not change as obviously as they did in the sequencing results.

Coexpression networks, including the seven most significantly expressed lncRNAs and other genes, were constructed to explore potential target genes for these lncRNAs (Figure S1).

Figure 2D indicates that most of the differentially expressed genes were enriched in the metabolic pathway category. Thus, we focused our attention on the modulation of glucose metabolism during cetuximab resistance. Levels of glucose metabolism‐related genes were detected. In H508/CR cells, hypoxia inducible factor 1 subunit alpha (*HIF1A*), hexokinase 1 (*HK1*), hexokinase 2 (*HK2*), pyruvate dehydrogenase kinase 3 (*PDK3*), and aldehyde dehydrogenase 1 family member A3 (*ALDH1A3*) levels were elevated, while the expression of isocitrate dehydrogenase (NADP(+)) 2, mitochondrial (*IDH2*), which is involved in the tricarboxylic acid (TCA) cycle, was reduced (Figure 3B). Furthermore, H508/CR cells showed increased glucose consumption and lactate secretion relative to cetuximab‐sensitive cells (Figure 3C,D).

### 3.5 *LINC00973* inhibition attenuated cetuximab resistance and was associated with glucose metabolism

**Figure 4:**
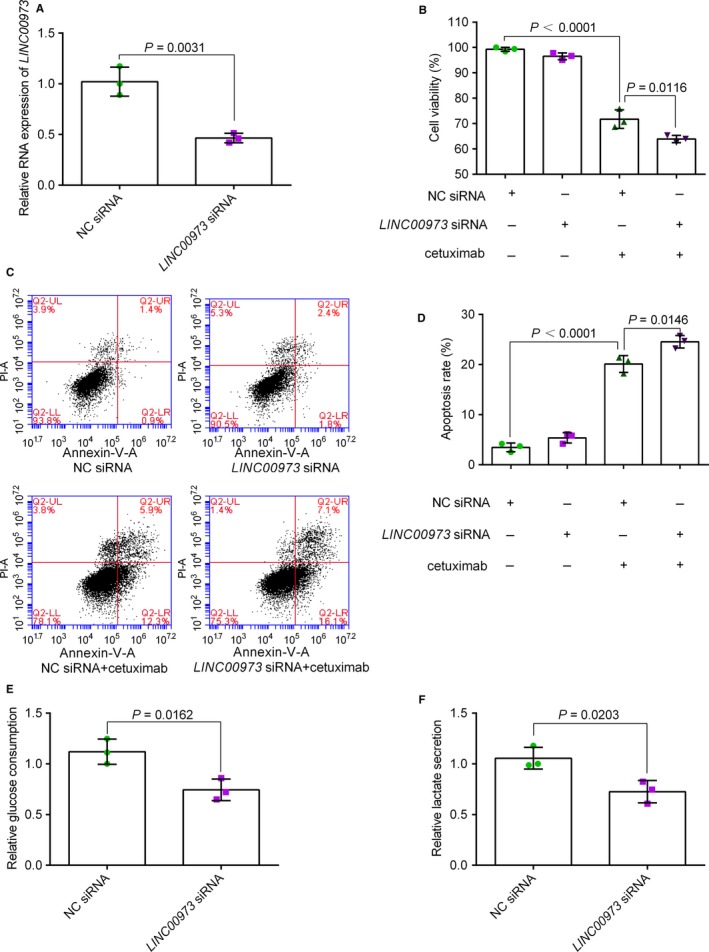
The effect of *LINCOO973* on cetuximab resistance. (A) H508/CR cells were prepared for reverse transcription‐quantitative polymerase chain reaction analysis following transient transfection with NC siRNA or *LINCOO973* siRNA. Unpaired Student's *t* tests were used in Figure 5A. The transfected cells were then treated with 10 μg/mL cetuximab for a further 72 h. Treated cells were harvested for a cell viability assay (B) and flow cytometry graphs are representative of three separate experiments (C). The apoptosis rates of three independent experiments were presented in D. Statistical comparisons were performed using two way ANOVA followed by Bonferroni posttests in Figure 5B and C. Glucose consumption (E) and secreted lactate levels (F) by *LINCOO973* siRNA transfected H508/CR cells in 72 h were shown as fold changes of NC siRNA transfected H508/CR cells. Unpaired Student's *t* tests were used in Figure 5E and F. NC, negative control; si, small interfering; PI, propidium iodide. Every test was carried out in triplicate. Data are presented as means ± SD

**Figure 5:**
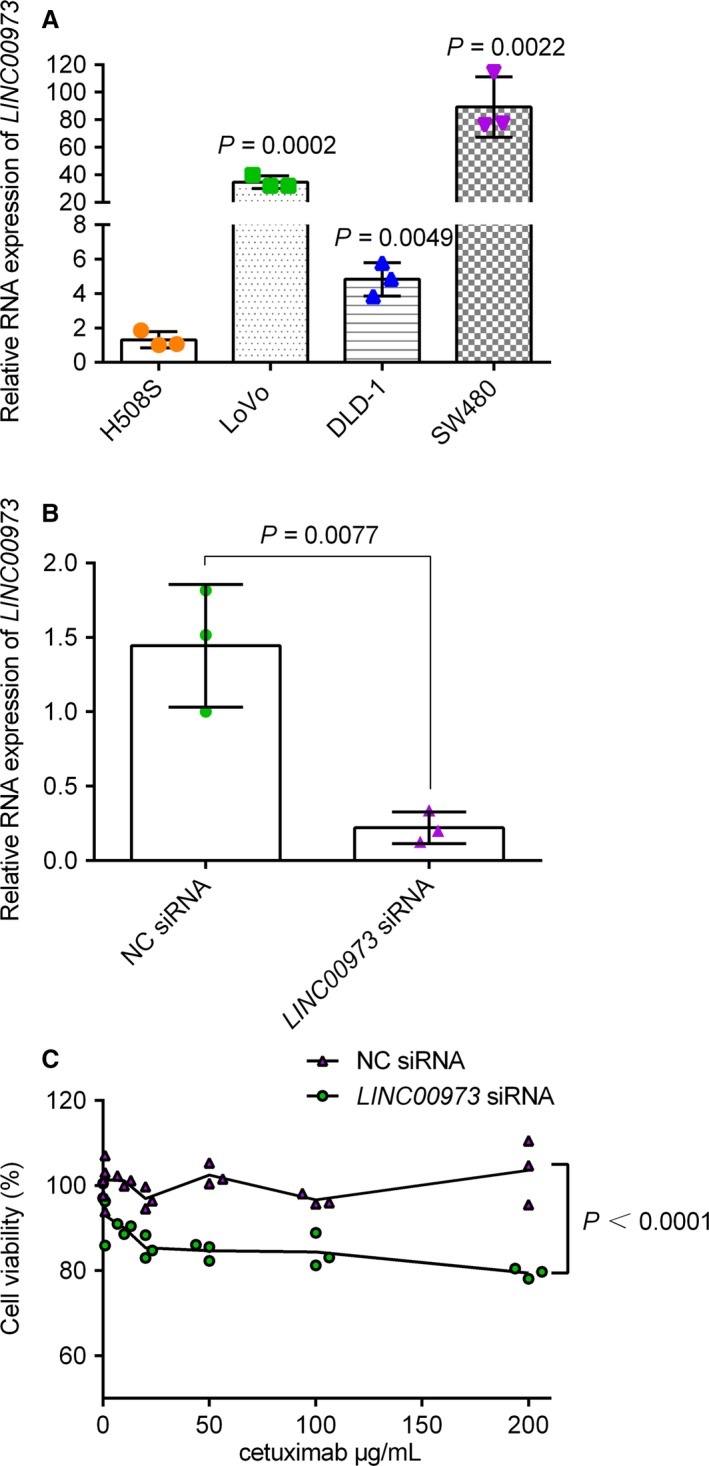
The effect of *LINCOO973* on cetuximab resistance. (A) Four cell lines were prepared for reverse transcription‐quantitative polymerase chain reaction analysis. *P‐*values were for comparison with H508S cells by unpaired Student's *t* test. (B) SW480 cells were transfected with NC siRNA or *LINCOO973* siRNA and then for qRT‐PCR analysis. Unpaired Student's *t* tests were used between two groups. (C) Cells were treated with indicated concentrations of cetuximab (1, 10, 20, 50, 100, 200 μg/mL) for 72 h. Cell viability was determined by CCK‐8 assay. Every CCK‐8 test was carried out in quadruplet. Data are mean ± SD for three independent experiments. Statistical comparisons were performed at each dose using two way ANOVA (group effect, *F*(1,28) = 109.2, *P* < 0.0001)

To determine whether these lncRNAs could affect cetuximab sensitivity, we chose the most significantly upregulated lncRNAs for further study. RNA‐FISH results further confirmed that the *LINC00973* level was increased in H508/CR cells (Figure S2). H508/CR cells were transfected with *LINC00973* siRNA or NC siRNA. After 48 hours of transfection, the levels of the lncRNAs were measured to test the transfection efficiency. This confirmed that the levels of the lncRNAs were decreased after transfection (Figure 4A). A CCK‐8 assay showed that silencing of *LINC00973* significantly increased the cetuximab sensitivity of H508/CR cells (Figure 4B). The cell viability of NC siRNA and cetuximab treatment group was 71.73 ± 3.66%, while in the *LINC00973* siRNA and cetuximab treatment group, cell viability decreased to 63.91 ± 1.43%. Simultaneously, as presented in Figure 4C,D, the flow cytometry analysis revealed that low *LINC00973* expression decreased cetuximab resistance by increasing cell apoptosis in H5058/CR cells (the flow cytometry gating strategy is shown in Figure S3). Under cetuximab stimulation, the *LINC00973* siRNA group exhibited increased apoptosis (24.53 ± 1.26%) compared with that in the NC siRNA transfection group (20.10 ± 1.68%). Furthermore, *LINC00973* inhibition decreased glucose consumption and lactate secretion to 74.37 ± 10.62% and 72.53 ± 10.98%, respectively (Figure 4E,F). In addition, we examined the levels of *LINC00973* in SW480, DLD‐1, and LoVo human CRC cell lines. Interestingly, all the three cell lines, which are cetuximab resistant, displayed higher expression of *LINC00973* than that in H508S cells (Figure 5A). We selected SW480 cells with the highest expression of *LINC00973* for siRNA transfection. A CCK‐8 assay demonstrated that *LINC00973* siRNA sensitized the SW480 cells to cetuximab (Figure 5B,C).

## 4 DISCUSSION

The combined application of cetuximab and conventional chemotherapy is a typical therapeutic regimen for patients with mCRC. However, primary and secondary resistance limits the further clinical application of cetuximab. Therefore, it is important to investigate potential strategies to improve the response of CRC to cetuximab. However, to date, very few reports have explored the relationship between lncRNAs and cetuximab resistance. Lu et al generated cetuximab‐resistant cells in three‐dimensional culture and used sequencing to reveal that no known genetic factors were related to cetuximab resistance. LncRNA *MIR100HG* and two embedded microRNAs, miR‐100 and miR‐125b, were overexpressed in cetuximab‐resistant cells. The two miRNAs synergistically inhibit five negative regulators of the Wnt/β‐catenin pathway, leading to increased Wnt signaling. Moreover Wnt inhibition in cetuximab‐resistant cells could increase cetuximab sensitivity.^15^ Mining in the GEO database showed that expression of lncRNA *POU5F1P4* was decreased in cetuximab‐resistant cells and tissues and its level was associated with the progression free survival of patients with mCRC. Targeting *POU5F1P4* could change the cetuximab sensitivity of CRC cells.^16^

In the present study, we used H508/CR cells to study cetuximab resistance mechanisms. No known genetic events that were linked to cetuximab resistance were identified in the resistant cells. We evaluated the levels of the most significant lncRNAs. The quantitative PCR results were basically consistent with RNA‐Seq data. Some of these lncRNAs have not been reported before. Hundreds of lncRNAs may be involved in cetuximab resistance by cis‐ or trans‐ regulation of tens of thousands of genes.

The RNA‐Seq data offer new information about the potential regulatory mechanism of cetuximab in CRC. Our results indicated that the function of these lncRNAs might be mediated through various signaling pathways, including metabolic pathways, focal adhesion, alcoholism, systemic lupus erythematosus, and pathways in cancer. Therefore, we hypothesized that these pathways may be potential therapeutic targets in cetuximab resistance. The Warburg effect explains that tumor cells produce a high glycolytic rate and lactate production without oxygen, which seems to be critical for the growth and invasion of tumor cells, metastasis, and prognosis.^17^ Glycolysis can facilitate tumor migration and invasion by producing excess lactic acid to form an acidic tumor microenvironment. In addition, it is also capable of producing biosynthetic precursors to inhibit apoptosis and promote tumor proliferation.^18^, ^19^ Aerobic glycolysis and mitochondrial energy metabolism are closely related to multidrug resistance. The regulation of energy metabolism could alter tumor growth and chemotherapy drug sensitivity.^20^, ^21^ Liver cancer cells acquired doxorubicin resistance by increasing mitochondrial energy metabolism and glycolysis.^22^ Compared with their parental cells HCC827, erlotinib‐resistant nonsmall cell lung cancer cells overexpressed glucose transporter 1 and displayed increased glucose uptake and glycolysis rate. The combined use of glucose deprivation and AKT or autophagy inhibitors could improve the sensitivity of acquired erlotinib for nonsmall cell lung cancer.^23^ In the present study, increased glucose consumption and lactate secretion in the cetuximab‐resistant cells suggested that glucose metabolism might be involved in cetuximab resistance. Thus, our study provided valuable insights for future research, which may be directed to confirm the exact relationship between cetuximab resistance, lncRNAs, and glucose metabolism in CRC.

A recent report demonstrated that *LINC00973* is most strongly and consistently increased upon treatment of colon cancer cell lines with 5‐fluorouracil, oxaliplatin, and irinotecan.^24^ We found that the expression of *LINC00973* was upregulated significantly in cetuximab‐resistant cells and knockdown of this lncRNA could ameliorate the resistance of H508 cells to cetuximab. Thus, the *LINC00973* expression level could be used as a prognostic tool to predict cetuximab resistance.

However, this study has some limitations. On one hand, we used only one resistant cell line to study the mechanisms of cetuximab resistance in vitro. Further investigations are needed to study the role of the lncRNAs. On the other hand, we demonstrated that *LINC00973* is closely related to cetuximab resistance; however, the detailed mechanisms require further research.

In conclusion, the high‐throughput transcriptome sequencing and bioinformatic analysis of cetuximab‐sensitive and resistant cell lines identified clinically relevant, epigenetic causes of cetuximab resistance and provided potential therapeutic targets for future research into CRC.
