# Supplementary figures and images for: Long noncoding RNA and mRNA profiling in cetuximab‐resistant colorectal cancer cells by RNA sequencing analysis
Source: Cancer Med. 2019 Mar 7;8(4):1641–51. doi: 10.1002/cam4.2004 (PMC6488152; doi:10.1002/cam4.2004)

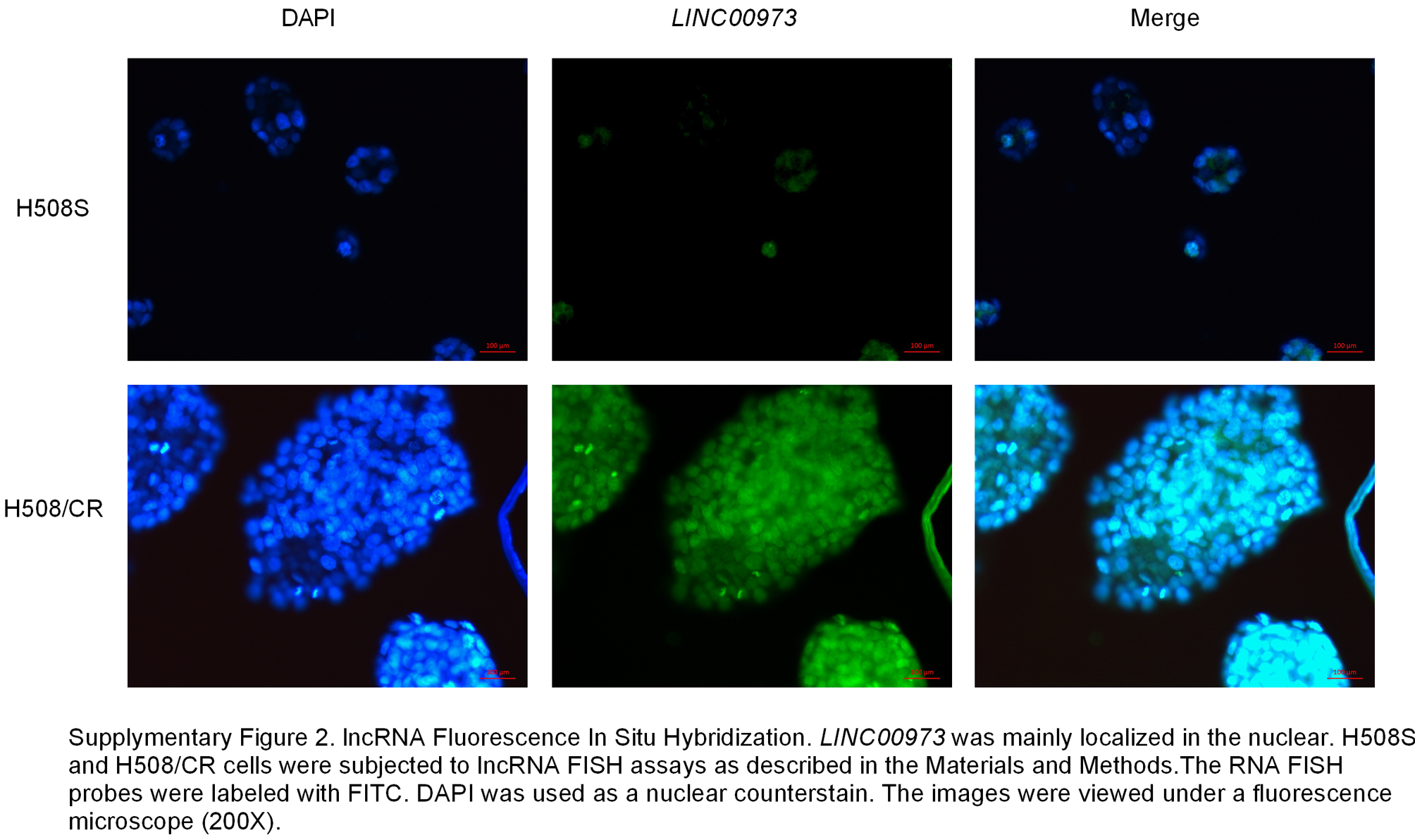

Supplement: Supplementary file 2 [file CAM4-8-1641-s002.tif]
