# Supplementary material for: Long noncoding RNA and mRNA profiling in cetuximab‐resistant colorectal cancer cells by RNA sequencing analysis
Source: Cancer Med. 2019 Mar 7;8(4):1641–51. doi: 10.1002/cam4.2004 (PMC6488152; doi:10.1002/cam4.2004)
Supplement: Supplementary file 4 [file CAM4-8-1641-s004.docx]

Table S1 Primers for lncRNAs and mRNAs

| Name | Primers (5’-3’) | Obtained Ct values |
| --- | --- | --- |
| *LINC00675* | F: ATCTCCTGCGAGCACATATCAA | 26.64-30.50 |
|  | R: CAACTGGTCAGTGTCAAAGGGTAG |  |
| *LINC01133* | F: TTTTGGTTTGAGGGCATAGGG | 26.21-28.14 |
|  | R: CAACAGCATTGACGAGACACATTT |  |
| *MRPL23-AS1* | F: GAGGGTCAAAGGCCACTCTG | 27.84-28.21 |
|  | R: GAAAACAAACACTTACTGGCTCTG |  |
| *LINC00973* | F:TGCTAGGCACGACTTCTGGT | 27.40-31.90 |
|  | R: GGTTGACAGTGAGGGAATAATTG |  |
| *IGFL2-AS1* | F: TGACACTGAATGTAGAGGTCGTTG | 26.10-30.60 |
|  | R: GGGTTGACAGGGTAGAATCTGA |  |
| *LINC01564* | F:GTAACCTCTGTCTCACTCCTCCAC | 34.10-37.00 |
|  | R: CAGGGCTGAATATGTTTTCTGC |  |
| *LINC02474* | F: ACAGCATCTGCCTTTGACCA | 29.10-30.08 |
|  | R: TCTCCAGCACCCAGTTCTAGC |  |
| *AC104823.1* | F: GCAGAAGCACCCAGGTATGA | 26.66-30.41 |
|  | R: CCTTTCCAAGTCCAATCACTGTA |  |
| *AL136418.1* | F:CCTGTAGGGAAGCTGCAACTG | 21.93-25.35 |
|  | R:CTGACATTGTCTTACTCCTTGATGA |  |
| *HIF-1A* | F:ATCCATGTGACCATGAGGAAATG | 22.14-24.47 |
|  | R:TCGGCTAGTTAGGGTACACTTC |  |
| *HK 1* | F:GCTCTCCGATGAAACTCTCATAG | 23.87-25.40 |
|  | R: GGACCTTACGAATGTTGGCAA |  |
| *HK 2* | F:GAGCCACCACTCACCCTACT | 24.71-25.98 |
|  | R:CCAGGCATTCGGCAATGTG |  |
| *PDK 3* | F:CGCTCTCCATCAAACAATTCCT | 25.10-27.75 |
|  | R:CCACTGAAGGGCGGTTAAGTA |  |
| *ALDH1A3* | F:TGAATGGCACGAATCCAAGAG | 26.84-29.62 |
|  | R:CACGTCGGGCTTATCTCCT |  |
| *IDH2* | F:CCCGTATTATCTGGCAGTTCATC | 22.89-23.47 |
| *β-actin* | R:ATCAGTCTGGTCACGGTTTGG  F:GATGAGATTGGCATGGCTTT  R: CACCTTCACCGTTCCAGTTT-3 | 16.14-18.02 |
