# Supplementary material for: Long noncoding RNA and mRNA profiling in cetuximab‐resistant colorectal cancer cells by RNA sequencing analysis
Source: Cancer Med. 2019 Mar 7;8(4):1641–51. doi: 10.1002/cam4.2004 (PMC6488152; doi:10.1002/cam4.2004)
Supplement: Supplementary file 5 [file CAM4-8-1641-s005.docx]

Table S2. Target sequences of *LINC00973* siRNA

| Product name | Target sequences |
| --- | --- |
| Ribo^TM^ h-*LINC00973* Smart Silencer | CACGACTTCTGGTCATTTA  GCAGTGACTTCGAGTACCA |
|  | GGATCAGAACTTCTACCTA |
|  | CTGTGTTACTCCTTCGCATT  GATACATTTGCTAGGCACGA   \| AGAATCCAGATTTAGAGGTC \| \| --- \| |
