# Supplementary material for: Long noncoding RNA and mRNA profiling in cetuximab‐resistant colorectal cancer cells by RNA sequencing analysis
Source: Cancer Med. 2019 Mar 7;8(4):1641–51. doi: 10.1002/cam4.2004 (PMC6488152; doi:10.1002/cam4.2004)
Supplement: Supplementary file 6 [file CAM4-8-1641-s006.docx]

Table S3. *P* values caculated by Bonferroni’s multiple comparisons test in Figure 1.

| Bonferroni's multiple comparisons test | Mean Diff. | 95% CI of diff. | Significant? | Summary | Adjusted P Value |
| --- | --- | --- | --- | --- | --- |
| Control |  |  |  |  |  |
| H508S vs. H508/CR | -1.233 | -5.476 to 3.009 | No | ns | > 0.9999 |
| H508S vs. H508/CR after 8 weeks | 0.7 | -3.543 to 4.943 | No | ns | > 0.9999 |
|  |  |  |  |  |  |
| 1 μg/ml Cetuximab |  |  |  |  |  |
| H508S vs. H508/CR | -15.7 | -19.94 to -11.46 | Yes | **** | < 0.0001 |
| H508S vs. H508/CR after 8 weeks | -14.1 | -18.34 to -9.857 | Yes | **** | < 0.0001 |
|  |  |  |  |  |  |
| 10 μg/ml Cetuximab |  |  |  |  |  |
| H508S vs. H508/CR | -27.53 | -31.78 to -23.29 | Yes | **** | < 0.0001 |
| H508S vs. H508/CR after 8 weeks | -23.13 | -27.38 to -18.89 | Yes | **** | < 0.0001 |
|  |  |  |  |  |  |
| 20 μg/ml Cetuximab |  |  |  |  |  |
| H508S vs. H508/CR | -22.17 | -26.41 to -17.92 | Yes | **** | < 0.0001 |
| H508S vs. H508/CR after 8 weeks | -18.43 | -22.68 to -14.19 | Yes | **** | < 0.0001 |
|  |  |  |  |  |  |
| 50 μg/ml Cetuximab |  |  |  |  |  |
| H508S vs. H508/CR | -21.03 | -25.28 to -16.79 | Yes | **** | < 0.0001 |
| H508S vs. H508/CR after 8 weeks | -16.27 | -20.51 to -12.02 | Yes | **** | < 0.0001 |
|  |  |  |  |  |  |
| 100 μg/ml Cetuximab |  |  |  |  |  |
| H508S vs. H508/CR | -16.63 | -20.88 to -12.39 | Yes | **** | < 0.0001 |
| H508S vs. H508/CR after 8 weeks | -13.37 | -17.61 to -9.124 | Yes | **** | < 0.0001 |
|  |  |  |  |  |  |
| 200 μg/ml Cetuximab |  |  |  |  |  |
| H508S vs. H508/CR | -13.19 | -17.43 to -8.944 | Yes | **** | < 0.0001 |
| H508S vs. H508/CR after 8 weeks | -6.543 | -10.79 to -2.301 | Yes | ** | 0.0017 |
