# Supplementary material for: Long noncoding RNA and mRNA profiling in cetuximab‐resistant colorectal cancer cells by RNA sequencing analysis
Source: Cancer Med. 2019 Mar 7;8(4):1641–51. doi: 10.1002/cam4.2004 (PMC6488152; doi:10.1002/cam4.2004)
Supplement: Supplementary file 7 [file CAM4-8-1641-s007.docx]

| ncRNA | H508S1 | H508S2 | H508S3 | H508R1 | H508R2 | H508R3 | H508S1,H508S2,H508S3 | H508R1,H508R2,H508R3 | Fold_change | log2(Fold_change) | p-value | q-value |
| --- | --- | --- | --- | --- | --- | --- | --- | --- | --- | --- | --- | --- |
| *ENST00000589310.1* | 0 | 1 | 0 | 10 | 39 | 36 | 0.329501 | 23.462 | 71.20459 | 6.153898 | 2.55E-05 | 0.002702 |
| *NR_033957.2* | 0 | 1 | 0 | 30 | 16 | 9 | 0.329501 | 15.60853 | 47.37019 | 5.565908 | 0.000745 | 0.034635 |
| *NR_038977.1* | 1 | 0 | 0 | 8 | 16 | 25 | 0.370151 | 13.69727 | 37.00455 | 5.209631 | 0.000148 | 0.010522 |
| *NR_036581.1* | 1 | 0 | 4 | 82 | 74 | 73 | 2.087079 | 64.43112 | 30.87143 | 4.9482 | 5.97E-17 | 8.87E-14 |
| *ENST00000430184.1* | 1 | 0 | 0 | 16 | 19 | 6 | 0.370151 | 11.41254 | 30.83212 | 4.946362 | 0.000423 | 0.022568 |
| *NR_033807.2* | 18 | 9 | 6 | 395 | 465 | 315 | 12.20362 | 328.3642 | 26.90711 | 4.749915 | 1.85E-42 | 2.19E-38 |
| *ENST00000473756.1* | 1 | 1 | 8 | 104 | 214 | 84 | 4.133509 | 110.7752 | 26.79933 | 4.744125 | 2.26E-07 | 6.11E-05 |
| *NR_135234.1* | 1 | 1 | 8 | 127 | 141 | 122 | 4.133509 | 109.2653 | 26.43403 | 4.724324 | 3.67E-24 | 8.73E-21 |
| *ENST00000566733.1* | 3 | 4 | 3 | 77 | 97 | 101 | 3.716154 | 76.9906 | 20.71782 | 4.3728 | 3.75E-17 | 6.36E-14 |
| *NR_026975.1* | 11 | 1 | 6 | 138 | 181 | 183 | 6.976555 | 140.4277 | 20.12851 | 4.331169 | 7.24E-26 | 2.15E-22 |
| *NR_110013.1* | 0 | 4 | 0 | 22 | 28 | 36 | 1.318005 | 24.11324 | 18.29526 | 4.193398 | 1.82E-06 | 0.000333 |
| *ENST00000555282.1* | 1 | 3 | 1 | 20 | 54 | 30 | 1.787887 | 28.62848 | 16.01247 | 4.001124 | 1.19E-05 | 0.001567 |
| *ENST00000607036.1* | 0 | 4 | 0 | 26 | 33 | 14 | 1.318005 | 20.31709 | 15.41504 | 3.946266 | 2.05E-05 | 0.002318 |
| *ENST00000429998.2* | 0 | 3 | 0 | 22 | 14 | 17 | 0.988504 | 15.01242 | 15.18701 | 3.924766 | 0.000299 | 0.017712 |
| *ENST00000432258.1* | 2 | 3 | 3 | 40 | 47 | 70 | 3.016502 | 44.11582 | 14.62483 | 3.870348 | 9.62E-10 | 4.57E-07 |
| *ENST00000549742.1* | 1 | 0 | 1 | 7 | 16 | 18 | 0.799383 | 11.40242 | 14.26402 | 3.834309 | 0.001266 | 0.049684 |
| *NR_046848.1* | 0 | 9 | 2 | 73 | 49 | 43 | 3.823975 | 46.65004 | 12.19936 | 3.608733 | 1.08E-07 | 3.13E-05 |
| *NR_111988.1* | 4 | 2 | 5 | 45 | 51 | 71 | 4.285767 | 46.92406 | 10.94881 | 3.452703 | 1.12E-09 | 5.11E-07 |
| *ENST00000419027.1* | 1 | 0 | 3 | 27 | 18 | 18 | 1.657847 | 17.82066 | 10.74928 | 3.426168 | 0.000103 | 0.007908 |
| *ENST00000443993.1* | 2 | 1 | 2 | 24 | 29 | 21 | 1.928267 | 20.6788 | 10.72403 | 3.422776 | 3.72E-05 | 0.003628 |
| *NR_027397.1* | 3 | 3 | 0 | 18 | 31 | 30 | 2.098957 | 22.00688 | 10.48468 | 3.39021 | 3.73E-05 | 0.003628 |
